# Stick–Slip Delamination of Monolayer MoS_2_ from SiO_2_ under Ambient Humidity

**DOI:** 10.1021/acs.jpcc.6c02563

**Published:** 2026-07-31

**Authors:** Sebastiaan Haartsen, Pantelis Bampoulis

**Affiliations:** † Physics of Interfaces and Nanomaterials group, 3230 MESA+ Institute for Nanotechnology and University of Twente, PO Box 217, 7500AE Enschede, The Netherlands

## Abstract

Delamination of two-dimensional (2D) materials from substrates plays a key role in 2D material transfer processes and applications in tribology and printing. Here, we studied the delamination of monolayer MoS_2_ from a SiO_2_ substrate at ambient relative humidity, using atomic force microscopy and force–distance spectroscopy. In contrast to dry conditions, tip retraction at ambient humidity is dominated by capillary forces between the tip and MoS_2_ and exhibits an extended force plateau of about 10–15 nm that is decorated by oscillatory sawtooth features. These oscillations indicate a stick-and-slip propagation of a circular delamination front between the MoS_2_ and SiO_2_ during lifting of the membrane by the tip, when intercalation of water reduces MoS_2_–substrate adhesion. This stick–slip delamination process is consistent with depinning from a spatially heterogeneous adhesion landscape, likely influenced by the nanoscale roughness of SiO_2_.

## Graphical abstract

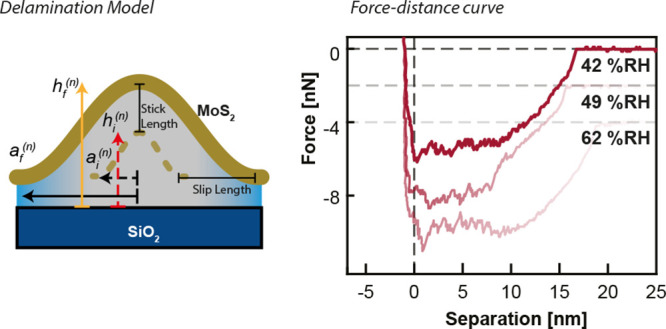

## I Introduction

Two-dimensional (2D) materials adhere to supporting substrates mainly via van der Waals forces.^1^ This makes their interfacial mechanics highly sensitive to surface characteristics, such as nanoscale roughness, adsorbates, and humidity.^2,3^ These interactions could control many key processes where 2D materials are involved, ranging from the standard fabrication and transfer of 2D materials and 2D material-based devices to their application and performance in tribological coatings and printing processes.^2–4^ In this regard, water condensation and intercalation under ambient conditions can introduce long-range adhesive forces that strongly modify interfacial interactions.^5,6^ Since the beginning of 2D material research delamination and blistering have been recognized as central failure phenomena in these systems.^7^ Most 2D material samples exfoliated from bulk crystals suffer from blisters, bubbles, wrinkles and other large deformations.^8–12^ Blister tests on graphene and related materials have been used to quantify adhesion energies.^13,14^ Furthermore, interfacial water has also been shown to substantially alter adhesion and enable complex delamination dynamics between the 2D material and the substrate.^15–22^ Unfortunately, and despite its importance in these processes, delamination of 2D crystals from supporting substrates remains poorly understood.

Here we probe the delamination of monolayer MoS_2_ on SiO_2_ using atomic force microscopy (AFM) force–distance measurements. At ambient relative humidity, we find a transition from a short-range pull-off regime that is dominated by direct adhesion to a regime characterized by long-range attraction and oscillatory plateaus that are consistent with capillary-driven membrane lifting and delamination crack advance. A stretching-dominated membrane model with a phenomenological stick–slip adhesion criterion reproduces the observed force oscillations and the stepwise evolution of the apparent blister radius.

## II Methods

The supporting substrate is freshly cut from a silicon wafer with an approximately 300 nm thermally grown SiO_2 layer_. We fabricated molybdenum-disulfide (MoS_2_) monolayers by mechanical exfoliation from a bulk crystal (purchased from HQ graphene, 99.995% purity). We used tweezers and scotch tape to carefully exfoliate flakes of MoS_2_, which were deposited on the SiO_2_–substrate without the use of polymers. Van-der-Waals interaction between MoS_2_ and SiO_2_ result in deposition of 2D material on the supporting substrate. MoS_2_ flakes on mica where deposited similarly to the silicon crystals. The mica was freshly cleaved beforehand. The flakes were thereafter identified with optical microscopy and their thickness was characterized with atomic AFM. Although the yield is quite low, the flakes have no polymer residue, which can otherwise influence the properties of our AFM-probe during scanning.^23^

AFM measurements are performed in a humidity-controlled environment using the Bruker Dimension Icon AFM. The sample is protected from the outside environment with a silicone ring, which encapsulates the sample along with the AFM-probe, creating a closed-off environment during measurements. A gas inlet and outlet allow us to control the humidity in situ, using a mixture of humid air and pure N_2_-gas. The relative humidity is measured at two locations using two SHT75 humidity and temperature sensors (accuracy ± 1.8% RH). One sensor is placed directly after mixing, and the other is placed at the outlet of the humidity cell. The gas flow is kept constant and low to ensure that the measurement is not affected by the gas flow. Measurements are performed 10 min after a humidity change to allow the system to stabilize.

Force–distance curves are obtained from 10 locations spread on the monolayer and supporting substrate. All force–distance curves were captured at the same peak force (2 nN) and length (60 nm). The silicon probes (PPP-FMR, Nanosensors^TM^) are calibrated on a hard sapphire surface before measurements. The spring constant of the probe (2.087 N/m) was calibrated before the measurements using the resonance frequency and thermal noise.^24^ The ramp frequency was 1 Hz, with 500 ms each for both approach and retraction with a constant tip speed of 120 nm/s. Local adhesion-force maps were obtained from force-distance measurements. At each measurement point, the adhesion force was defined as the magnitude of the most negative force measured on the retraction branch, corresponding to the pull-off force between the AFM probe and the exposed sample surface. For measurements on supported MoS_2_, this quantity therefore represents the local tip–MoS_2_ interaction and it is a proxy to the work of separation of the buried MoS_2_–substrate interface.

## III Results and Discussion

Figure 1a shows a topography image of a MoS_2_ flake on SiO_2_. Force–distance curves recorded on the MoS_2_ flake as the environmental relative humidity (RH) is increased indicate a transition in the interaction regime. At low RH (7% RH), the retract curve shown in Figure 1b exhibits a single pull-off event. This is characteristic of van der Waals adhesion coupled with minor capillary contributions between the AFM tip and the MoS_2_ monolayer. The sharp pull-off indicates that despite the tip interactions with the MoS_2_ membrane during the retract motion, the MoS_2_ monolayer remains adhered to the substrate. In this regime, the absence of long-range attraction implies that the formed liquid bridge is either negligible or ruptures at very small separations. At the same RH, a line profile, Figure 1c, across the MoS_2_ flake shows that the apparent height of the MoS_2_ monolayer is about 1.1–1.3 nm.^25^ This is larger than the intrinsic thickness expected for a single layer (about 0.62 nm^26–28^ ). This increase is attributed to a thin layer of interfacial water trapped between MoS_2_ and the hydrophilic SiO_2_ substrate,^6,22,29,30^ consistent with prior reports on 2D materials deposited on SiO_2_ and other hydrophilic substrates at ambient conditions.^4,31–34^ The yellow line profile in Figure 1c recorded at the same location and at 45% RH, shows an additional height increase of 0.3–0.6 nm, indicating further water intercalation at the interface.

**Figure 1:**
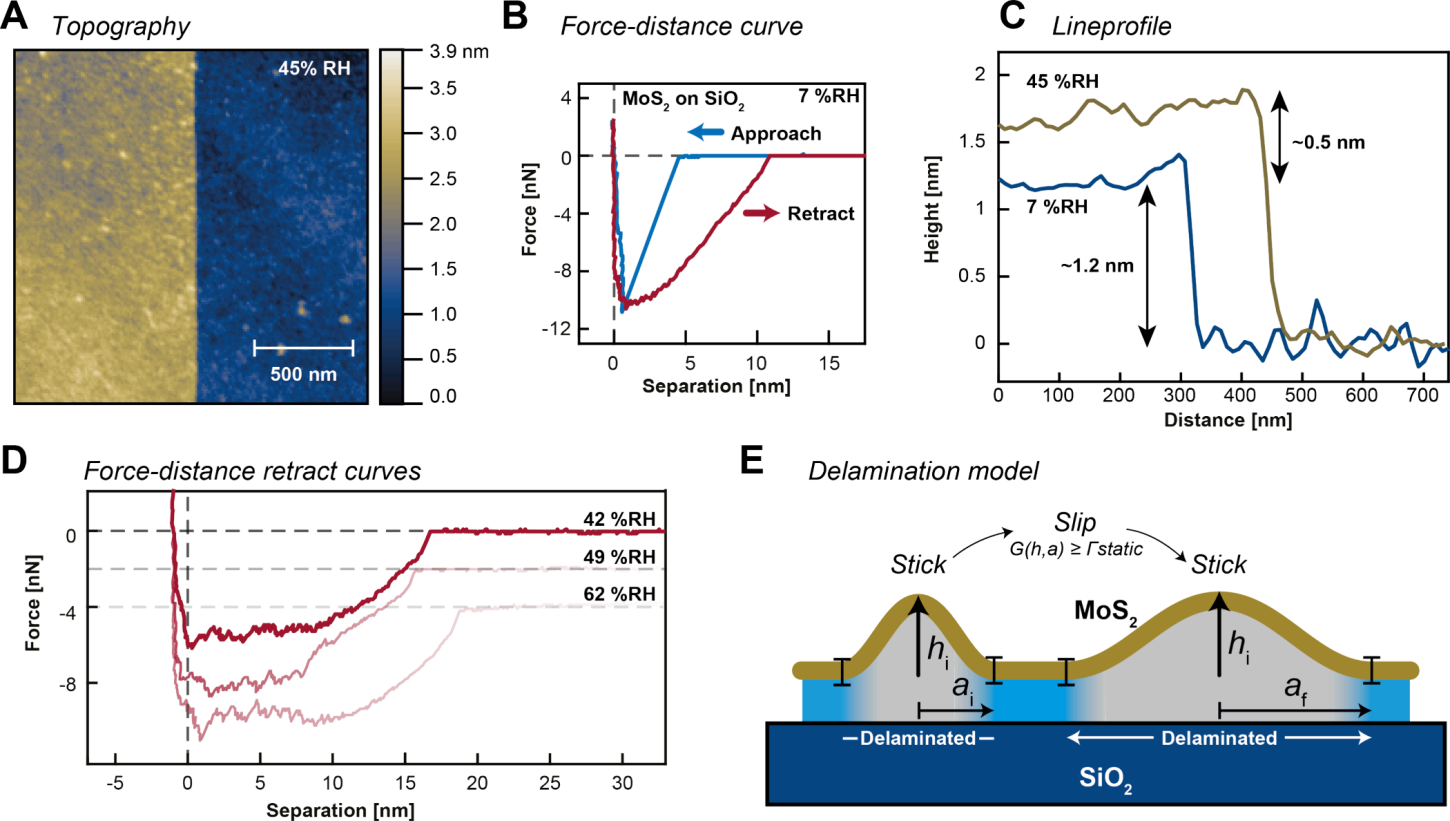
(a) AFM topography of a MoS_2_ monolayer on SiO_2_. (b) Retract curve at low RH (∼7% RH) showing single pull-off dominated by van der Waals adhesion. (c) Line profile showing the apparent monolayer height and increased thickness due to interfacial water. (d) Retract curves at ambient RH (42% RH to 62% RH) showing long-range attraction and oscillatory plateau. (e) A schematic illustrating the stochastic advancement of the delamination from $a_{i}^{\left(n\right)}$ to $a_{f}^{\left(n\right)}$ .

In contrast, the force–distance curves recorded at higher RH reveal a different and more complex behavior. Figure 1d shows multiple characteristic retract curves between 42% RH and 62% RH exhibiting a long-range attractive tail along with a force plateau with superimposed oscillations prior to detachment. These observations indicate the onset of a coupled capillary-membrane-fracture regime. The reduction of long-range attraction beyond the plateau is consistent with the elongation and rupture of a nanoscale capillary bridge between the tip and the MoS_2_ surface under ambient humidity conditions.^5,35–38^

The increase in the water content in both the tip-MoS_2_ and MoS_2_–SiO_2_ interfaces should not only increase the capillary interactions but also weaken the van der Waals coupling of the MoS_2_ to the substrate. The decrease in the MoS_2_/SiO_2_ adhesion and increase in the tip-MoS_2_ adhesion are what lead to the complex force–distance curves in Figure 1d. The capillary bridge formed between the AFM tip and the MoS_2_ surface acts as an external load that pulls the membrane upward during retraction. If the capillary-induced lifting generates an energy-release rate that exceeds the local work of separation of the MoS_2_/SiO_2_ interface, the membrane can locally delaminate. As the MoS_2_ lifts from the SiO_2_ surface it forms a bulged, delaminated region beneath the tip, i.e., a circular blister. Outside this region, the MoS_2_ remains adhered, producing a geometry equivalent to a point load acting on a circular blister that is bounded by a moving delamination front.^7,8,14,39^

Because the bending rigidity of the MoS_2_ monolayer is negligible compared to its in-plane stiffness,^9–11,40^ the mechanical response should be mainly governed by stretching of the membrane rather than bending. As the tip retracts further, the central deflection increases and the membrane stores elastic energy through in-plane strain. When the stored elastic energy becomes sufficient to overcome the local adhesion barrier, a circular crack front advances suddenly to a new position and increases the blister radius. The increase in delaminated area relaxes the membrane strain, leading to an immediate drop in force even though the tip position changes only minimally. After the slip event, the front becomes pinned again at a new position, initiating the next cycle, and producing the observed stick–slip oscillations in the force–distance curves in Figure 1d. We note here that the intercalated water at the MoS_2_/SiO_2_ interface plays a crucial role in enabling this instability. This is because it weakens the coupling of the MoS_2_ to the substrate. The mechanism of stick and slip described above is shown schematically in Figure 1e. During stick, the delamination front is pinned and the blister radius remains approximately constant. During a slip event *n*, the front advances from an initial radius $a_{i}^{\left(n\right)}$ to a new radius $a_{f}^{\left(n\right)}$ , with $a_{f}^{\left(n\right)}>a_{i}^{\left(n\right)}$ . The radial increment, $L_{slip}^{\left(n\right)}=\Delta a^{\left(n\right)}=a_{f}^{\left(n\right)}-a_{i}^{\left(n\right)}$ , is defined here as the slip length. In the schematics, h denotes the effective membrane-lifting coordinate extracted from the force–distance curve.

To understand this behavior in more detail, we model the tip-MoS_2_/SiO_2_ system as an atomically thin membrane adhered to a rigid substrate and pulled upward at its center by the AFM tip during retraction. We treat the delaminated region as a circular patch, with *a*, the radius of the circular edge where the membrane detaches from the substrate. The region *r* < *a* is approximated as a freestanding, clamped circular membrane subjected to a central point load at *r* = 0, while the membrane–substrate adhesion is represented energetically through a fracture criterion that governs the evolution of *a*. We assume axisymmetry, so that the force, deflection, and delamination front are the same in every azimuthal direction and the crack front remains circular. This is the same idealized geometry treated by Jin^41^ for a Föppl-Hencky membrane under a concentrated central force. In our case, Jin’s solution is not used as an exact description of the full AFM geometry, but as the local constitutive law for the restoring force of the lifted membrane.^42^ In this minimal model, we neglect residual pretension and bending contributions, and only assume that the mechanics are stretching-dominated. Furthermore, a complete thermodynamic treatment of our system should also include the capillary free energy of the tip-water-MoS_2_ meniscus and the elastic energy stored in the AFM cantilever. However, in our minimal model these contributions are not included.

Following Jin,^41^ the exact wrinkle-free point-load solution for a clamped circular membrane can be written as

$${\left(\frac{w_{0}\left(z\right)}{t}\right)}^{3}=g\left(\nu \right)F,,F=\frac{4a^{2}P_{0}}{\pi Et^{4}}$$

where *w*_0_ is the central membrane deflection, *t* is the membrane thickness, *a* is the membrane radius, *E* is Young’s modulus, ν is Poisson’s ratio, *P*_0_ is the applied central point load, and *g*(ν) is the Poisson-ratio-dependent dimensionless factor from the exact/parametric point-load membrane solution.^41,43^ Solving for *P*_0_ gives

$$P_{0}=\frac{\pi Et}{4g\left(\nu \right)}\frac{{w_{0}}^{3}}{a^{2}}$$

Defining the two-dimensional tensile modulus *E*_2*D*_ = *Et*, the positive elastic restoring-force magnitude of the lifted membrane becomes

$$F_{bulge}\left(w_{0},a\right)=\frac{\pi E_{2D}}{4g\left(\nu \right)}\frac{{w_{0}}^{3}}{a^{2}}$$

Here, *w*_0_ is the central out-of-plane deflection of the membrane. To connect the model to the measured retract curve (separation distance *z*), we use the effective approximation

$$w_{0}\approx h\approx z-z_{0}$$

within the oscillatory plateau region only, where *z*_0_ denotes the onset of membrane lifting. Adhesion between the substrate and the membrane enters the model through the evolution of *a*(*z*), which changes the membrane stiffness and therefore the measured force through the *a* ^–2^ dependence of *F*_bulge_.

The external force driving delamination is taken to be dominated by the capillary bridge between the AFM tip and the membrane. This bridge acts as an attractive load applied effectively at the membrane apex. As the tip retracts, the bridge elongates, and the attraction gradually decays, producing the extended attractive region of the retract curve. To represent this term in a minimal way, while allowing for possible contributions from other interactions such as van der Waals or electrostatic forces, we write the capillary-force magnitude empirically as

$$F_{cap}\left(w_{0}\right)=\{\begin{array}{ll} F_{cap,0}\exp\left(-\frac{w_{0}}{L_{cap}}\right), & w_{0}\leq w_{R}\\ 0, & w_{0}>w_{R} \end{array}$$

where *F*_cap,0_ is the capillary-force magnitude at *w*_0_ = 0, *L*_cap_ is a decay length, and *w*_*R*_ is the effective rupture displacement at which the meniscus breaks. This term is used only as a minimal interpolation to describe the long-range attractive force (typically due to capillary forces) observed during tip retraction under humid conditions.^44,45^

Using the AFM sign convention in which attraction is negative, the measured AFM retract force is written as

$$F_{meas}\left(w_{0}\right)=F_{bulge}\left(w_{0},a\left(w_{0}\right)\right)-F_{cap}\left(w_{0}\right)$$

During the oscillatory plateau, the measured force (*F*_meas_) remains attractive because *F*_cap_ > *F*_bulge_, so that *F*_meas_ < 0. As the capillary bridge weakens during retraction, the force approaches zero, and after meniscus rupture, the attractive term vanishes. We note here that the capillary bridge is treated as the external loading mechanism and its detailed surface-energy contribution is not included.

For a fixed delamination radius *a*, the elastic strain energy stored in the membrane is obtained by integrating the point-load relation:^41^

$$U\left(w_{0},a\right)=\int_{0}^{w_{0}}F_{bulge}\left(w_{0}^{\prime },a\right)\;dw_{0}^{\prime }=\frac{\pi E_{2D}}{16g\left(\nu \right)}\frac{{w_{0}}^{4}}{a^{2}}$$

We treat the membrane delamination phenomenologically as a crack that grows only when there is sufficient mechanical energy to create an additional separated interface. As the front moves outward by a small amount, *da*, the delaminated area increases and the elastic energy stored in the lifted membrane decreases. The energy-release rate *G* (units *J*/*m* ^2^) is defined as

$$G=-\frac{\partial U}{\partial A}$$

where *A* is the delaminated area. When *G* is smaller than the interface adhesion energy Γ, there is not enough energy for the crack to grow. When *G* becomes equal to or larger than Γ, the crack can advance.^42,46,47^

For a circular delamination front, the delaminated area is *A* = *πa* ^2^, so $\frac{dA}{da}=2\pi a$ . Holding *w*_0_ fixed while differentiating with respect to *a*, the driving force becomes

$$G\left(w_{0},a\right)=-\frac{\partial U}{\partial A}=-\frac{1}{2\pi a}\frac{\partial U}{\partial a}=\frac{E_{2D}}{16g\left(\nu \right)}\frac{{w_{0}}^{4}}{a^{4}}$$

However, the experiments show oscillations, which suggest a stick–slip process. We implement this phenomenologically by alternating between stick (a pinned crack front) and slip (a sudden outward jump of the delamination front).

We define Γ as the effective work of separation per unit area of the MoS_2_/SiO_2_ interface, similar to the adhesive interaction between two interfaces.^48^ Using the Griffith criterion, crack growth is allowed when *G* ≥ Γ. To produce stick–slip behavior, we distinguish between two thresholds: Γ_static,event_, the trigger threshold required to initiate motion of the front, and Γ_dynamic_, the lower effective resistance during propagation. At a given retraction *z*, equivalently at a given effective central deflection *w*_0_ ≈ *z* – *z*_0_, if:

$$G\left(w_{0},a\right)<\Gamma_{static,event}$$

then *a* remains constant and the system is pinned. When the driving force reaches the event threshold,

$$G\left(w_{0},a\right)\geq \Gamma_{static,event}$$

the system slips, and the front advances outward instantaneously. During such an event, *a* increases suddenly, and because of that *G* ∝ 1/*a* ^4^, and *F*_bulge_ ∝ 1/*a* ^2^, decrease immediately. This causes a drop in the measured force at nearly the same *z*. During propagation (slip event), we assume that the effective fracture resistance drops from the static level to the lower dynamic value Γ_dynamic_. The arrest condition is therefore

$$G\left(w_{0},a_{f}\right)=\Gamma_{dynamic},,\Gamma_{static,event}>\Gamma_{dynamic}$$

Solving for the arrested radius gives

$$a_{f}={\left(\frac{E_{2D}{w_{0}}^{4}}{16g\left(\nu \right)\Gamma_{dynamic}}\right)}^{1/4}$$

This produces discrete jumps in *a* and discrete drops in force, i.e., ’stick–slip’ motion. To represent event-to-event variability, we randomize the static threshold as

$$\Gamma_{static,event}=\max\left[\Gamma_{dynamic}+\Delta \Gamma_{\min},\Gamma_{static}\left(1+\sigma_{pin}\xi \right)\right]$$

where ξ∼N(0,1) is a random number drawn from a standard normal distribution and σ_*pin*_ is a dimensionless disorder amplitude that sets the size of the event-to-event fluctuations, and ΔΓ_min_ > 0 ensures that Γ_static,event_ > Γ_dynamic_ for every event. If σ_*pin*_ is zero, there is no randomness.^49,50^

The model described above produces the results shown in Figure 2a,b,c, demonstrating the oscillatory nature of the force–distance curves and the stepwise change of the bulge radius associated with a change of the effective blister opening angle $\left(\theta \left(z\right)=2\tan^{-1}\left(\frac{w_{0}\left(z\right)}{a\left(z\right)}\right)\right)$ . We note that here θ represents the apparent opening angle of an idealized blister cross-section, not the microscopic contact angle at the delamination front. The blister radius *a*(*z*) in the experimental results is a model-derived effective blister radius within the circular-blister approximation, and it is obtained by inverting the membrane relation. This radius should be interpreted as an apparent radius because it depends on the empirical capillary-force correction, the assumption *w*_0_ ≈ *z* – *z*_0_, and the stretching-dominated membrane approximation. The results are shown in Figure 2d,e,f, demonstrating that indeed *a* is changing in a stepwise fashion and that the effective blister angle shows a sawtooth shape in our experiments, in line with the stick and slip model.

**Figure 2:**
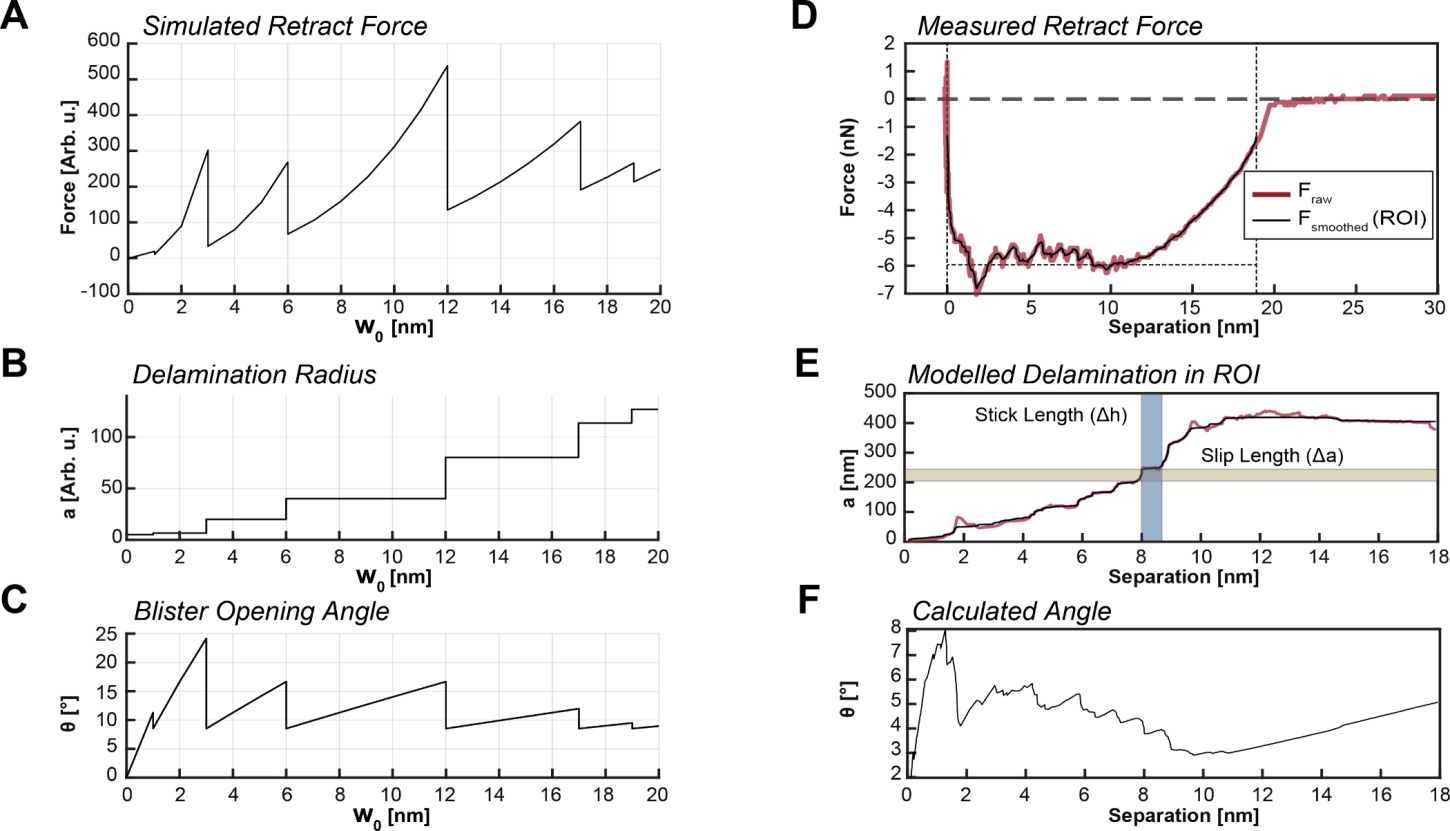
(a–c) Simulated force–distance curves, blister radius evolution, and effective blister opening angle from the membrane-fracture model. (d–f) Corresponding quantities extracted from experimental data showing stepwise increases in delamination radius and sawtooth variation in the apparent blister angle in the region of interest (ROI).

In Figure 3, we quantify the statistics of the stick–slip events extracted from force–distance curves recorded between 42% RH and 62% RH. For each event, we extract the stick length, *L*_stick_ = Δ*h*, the slip length, *L*_slip_ = Δ*a*, and the separation distance of the event onset, *z*_event_, at which the slip occurs. The stick length describes the increment in effective lifting coordinate accumulated while the delamination front remains pinned, whereas the slip length describes the radial advance of the delamination front during depinning. The distribution of stick lengths is skewed and shows a maximum around 0.5–1 nm. This indicates that most pinned segments are short: only a small additional lift is required before the local depinning threshold is reached. Larger stick lengths occur less frequently, consistent with event-to-event variability in local pinning barriers. The slip-length distribution is even more skewed, with most events concentrated around 25–50 nm and a long tail toward larger radial advances. This broad distribution shows that the front does not advance by a characteristic step size, but instead samples a heterogeneous adhesion landscape with variable local arrest positions. The distribution of event onsets, *z*_event_, is broader and less sharp than the stick- and slip-length distributions. Nevertheless, most events occur preferentially below about 15 nm separation, after which the occurrence probability decays. The decrease in event occurrence at larger *z*_event_ is consistent with the gradual weakening of the capillary bridge during retraction. At small to intermediate separations, the capillary attraction remains sufficient to lift the membrane and drive intermittent depinning of the delamination front. At larger separations, the bridge elongates and the attractive force decreases, reducing the ability of the tip to sustain further membrane lifting before total detachment. Nevertheless, the long plateau with the oscillatory behavior due to stick–slip events suggests that the process is governed by disorder at the interface rather than a deterministic process. This is consistent with depinning dynamics of an elastic interface moving through a heterogeneous energy landscape, characteristic of crack propagation in disordered systems.^49–51^ We note here that several alternative origins of the sawtooth-like force signals were also considered, including direct rupture of the tip–sample capillary bridge, cantilever instability, instrumental noise, contamination, and repeated tip–sample contact events. These explanations are unlikely to account for the complete response because the oscillatory plateau is observed reproducibly for MoS_2_/SiO_2_ under humid conditions, is suppressed at low humidity, and is largely absent for MoS_2_/mica under comparable conditions. Moreover, the extended plateau with repeated force drops is consistent with progressive membrane lifting and intermittent delamination, rather than with a single capillary rupture event.

**Figure 3:**
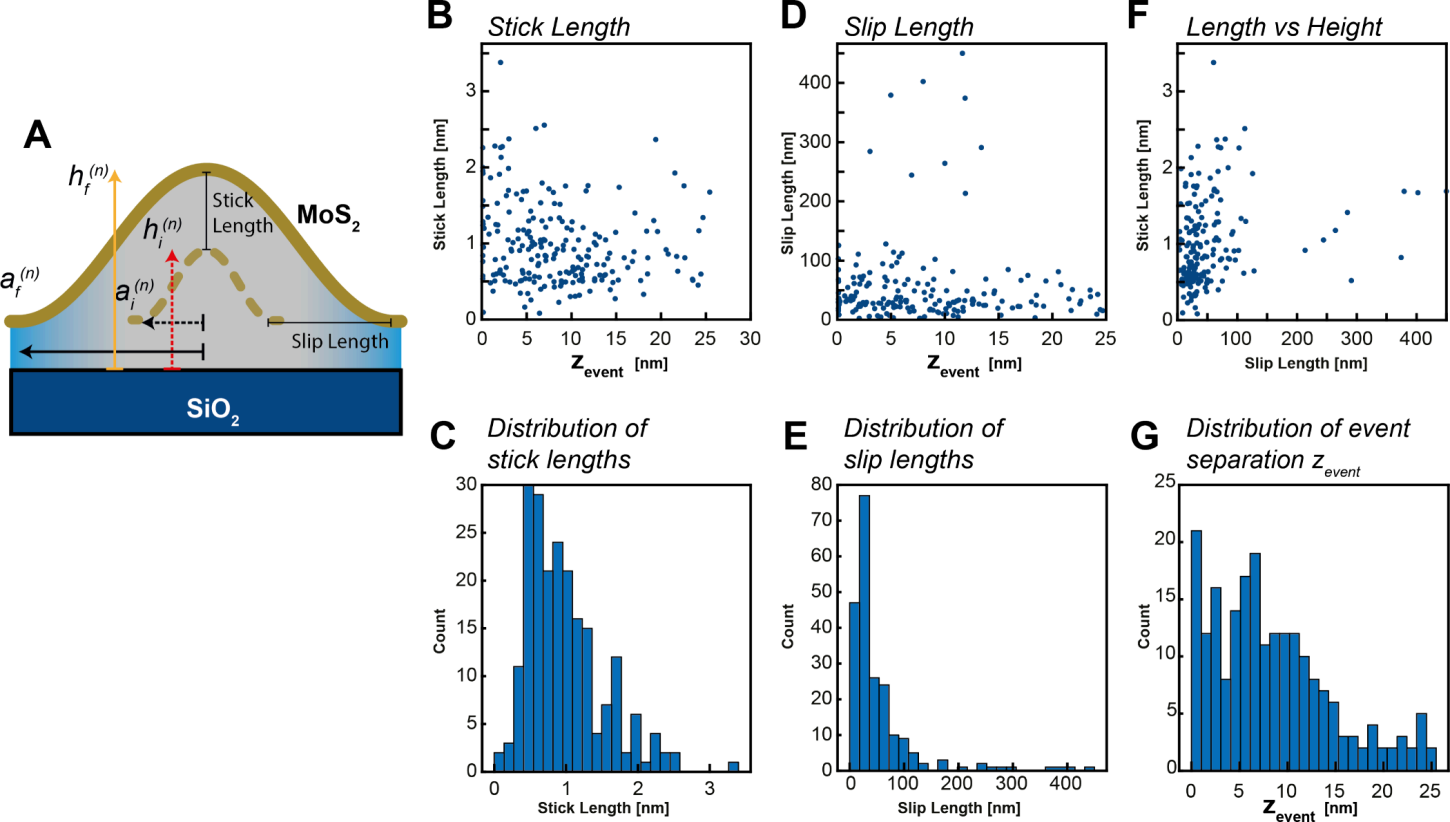
Statistics of stick–slip events. (a) Schematic of a representative stick–slip event, defining the extracted quantities. The stick length, *L*_stick_ = Δ*h*, is the increment in the effective lifting coordinate accumulated while the delamination front remains pinned. The slip length, *L*_slip_ = Δ*a*, is the radial advance of the delamination front during the subsequent slip event. (b) Stick length as a function of event separation. (c) Distribution of stick lengths. (d) Slip length as a function of event separation. (e) Distribution of slip lengths. (f) Stick length versus slip length. (g) Distribution of the separation onset, *z*_*event*_, at which stick–slip events occur.

We hypothesize that the nanoscale roughness of the SiO_2_ substrate contributes to the disorder in the effective adhesion landscape. Figure 4a compares the height distributions of SiO_2_ and mica, together with the corresponding supported MoS_2_ monolayers. The SiO_2_ substrate shows a broader height distribution than mica, indicating a larger nanoscale roughness. This roughness is partially reflected in the supported MoS_2_ layer, for which the height distribution on SiO_2_ remains broader than that on mica. The adhesion measurements show the same trend in the local interaction strength. Figure 4b shows that the adhesion distribution measured on MoS_2_/SiO_2_ is substantially broader than that measured on MoS_2_/mica. This indicates that the MoS_2_/SiO_2_ interface has a more heterogeneous local pull-off response, whereas the MoS_2_/mica interface is more spatially uniform. The combination of larger height variation and broader adhesion variation on SiO_2_ therefore supports the presence of a disordered adhesion landscape. This heterogeneous adhesion landscape provides a plausible origin for the stochastic stick–slip delamination observed on MoS_2_/SiO_2_. Local variations in adhesion modify the depinning threshold of the delamination front, so that some regions arrest the front while others allow sudden advance. In contrast, the smoother topography and narrower adhesion distribution on mica are consistent with stronger and more uniform coupling of MoS_2_ to the substrate, suppressing observable membrane lifting and stick–slip delamination in force–distance curves under comparable humidity conditions. This is also evident in Figure 4c, which shows the force–distance retract curves for MoS_2_ on mica at comparable humidities. Compared to Figure 1d, there are no noticeable stick–slip events.

**Figure 4:**
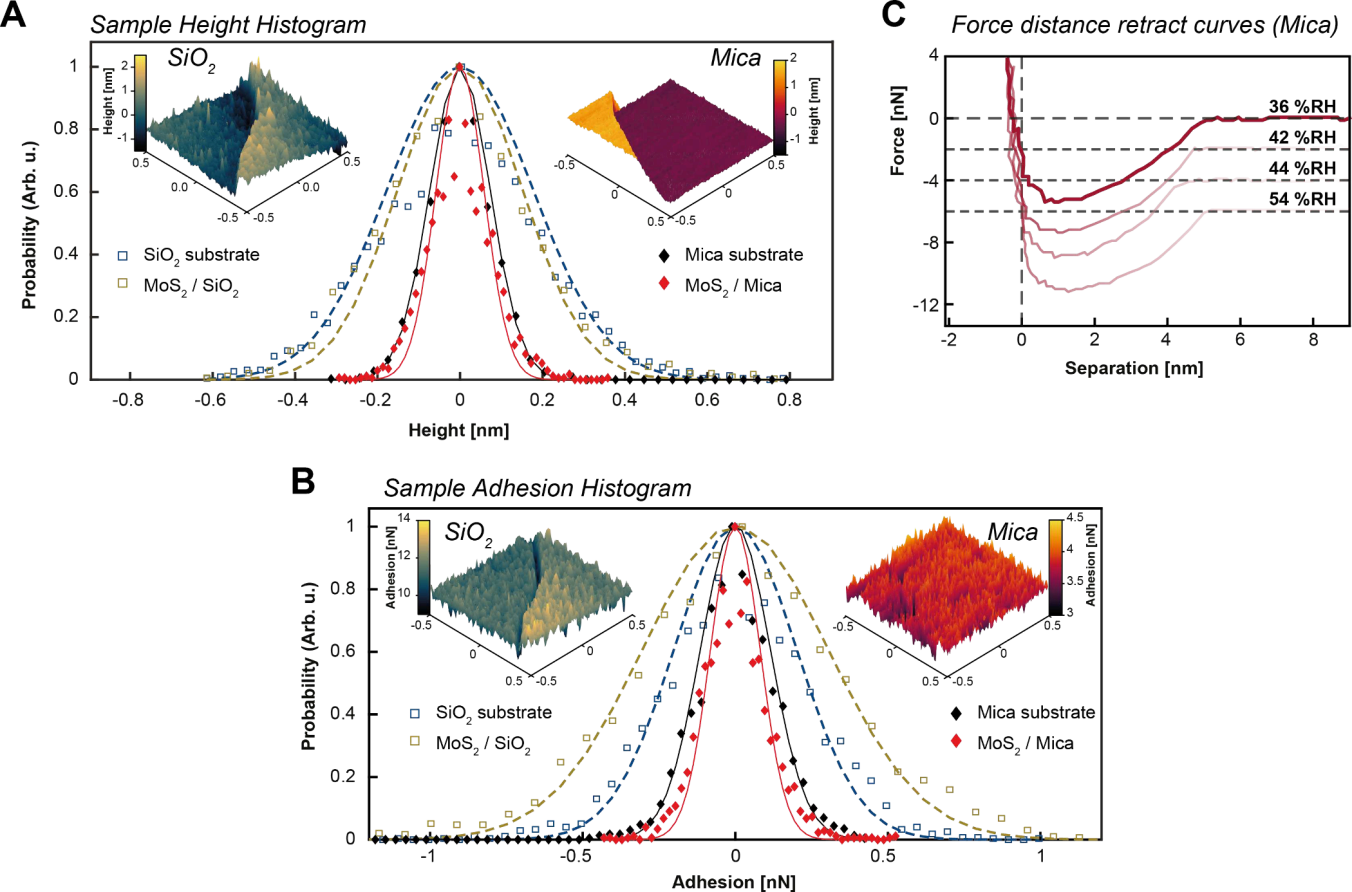
(a) Height distributions of the bare substrates and supported MoS_2_ monolayers, showing the larger nanoscale roughness of SiO_2_ compared with mica. Insets show representative topography maps. (b) Adhesion–force distributions measured on the same substrate systems. The adhesion distribution of MoS_2_/SiO_2_ is substantially broader than that of MoS_2_/mica. Insets show representative adhesion maps. The y-axes in (a) and (b) reflect normalized probabilities. The solid lines are guides to the eye. (c) Force–distance retract curves of MoS_2_ on mica at humidities comparable to Figure 1d, showing no pronounced stick–slip delamination plateau.

## IV Conclusion

We demonstrated that humidity fundamentally alters the delamination mechanics of monolayer MoS_2_ on SiO_2_ by introducing capillary forces and interfacial water that weaken membrane–substrate adhesion. Atomic force microscopy measurements reveal a transition from single pull-off events at low humidity to long-range attractive interactions with pronounced oscillations at ambient humidity. These oscillations are consistent with stick–slip propagation of a circular delamination front as a capillary bridge lifts the membrane while the stretching-dominated sheet depins from a spatially heterogeneous interface. Analysis of many stick–slip events has revealed that the delamination parameters vary widely and that the events are stochastic. Similar experiments on mica, which is smoother than SiO_2_, support the interpretation that substrate roughness contributes to the disordered adhesion landscape.
